# Ribosomal ambiguity (*ram*) mutations promote the open (off) to closed (on) transition and thereby increase miscoding

**DOI:** 10.1093/nar/gky1178

**Published:** 2018-11-22

**Authors:** Eric D Hoffer, Tatsuya Maehigashi, Kurt Fredrick, Christine M Dunham

**Affiliations:** 1 Department of Biochemistry and Emory Antibiotic Resistance Center, Emory University School of Medicine, Atlanta, GA 30322, USA; 2 Department of Microbiology and Center for RNA Biology, The Ohio State University, Columbus, OH 43210, USA

## Abstract

Decoding is thought to be governed by a conformational transition in the ribosome—open (off) to closed (on)—that occurs upon codon–anticodon pairing in the A site. Ribosomal ambiguity (*ram*) mutations increase miscoding and map to disparate regions, consistent with a role for ribosome dynamics in decoding, yet precisely how these mutations act has been unclear. Here, we solved crystal structures of 70S ribosomes harboring 16S *ram* mutations G299A and G347U in the absence A-site tRNA (A-tRNA) and in the presence of a near-cognate anticodon stem-loop (ASL). In the absence of an A-tRNA, each of the mutant ribosomes exhibits a partially closed (on) state. In the 70S-G347U structure, the 30S shoulder is rotated inward and intersubunit bridge B8 is disrupted. In the 70S-G299A structure, the 30S shoulder is rotated inward and decoding nucleotide G530 flips into the *anti* conformation. Both of these mutant ribosomes adopt the fully closed (on) conformation in the presence of near-cognate A-tRNA, just as they do with cognate A-tRNA. Thus, these *ram* mutations act by promoting the open (off) to closed (on) transition, albeit in somewhat distinct ways. This work reveals the functional importance of 30S shoulder rotation for productive aminoacylated-tRNA incorporation.

## INTRODUCTION

During protein synthesis, ribosomes select correct aminoacyl-tRNAs (aa-tRNAs) by monitoring the nucleotide (nt) pairing between the anticodon of the tRNA and the codon on the mRNA in the aminoacyl (A) site. Despite the large pool of near-cognate tRNA, the ribosome is highly accurate with an error rate on the order of 10^−3^ to 10^−5^ (1–4). High fidelity is achieved in part through a kinetic proofreading mechanism. GTP hydrolysis by EF-Tu effectively divides the decoding process into two stages, providing a second opportunity for rejection of incorrect aa-tRNA (5–7).

Aminoacyl-tRNA binds the ribosome as part of a ternary complex with EF-Tu and GTP (8). Initial binding, mediated by the interaction of 50S ribosomal proteins L7/12 with EF-Tu (9), is followed by sampling of the A-site codon by the tRNA. Codon-anticodon pairing in the 30S A site leads to activation of EF-Tu and GTP hydrolysis. The acceptor end of aa-tRNA then dissociates from EF-Tu and moves into the 50S A site, a step called accommodation. Once in the A/A site (indicating the tRNA position on the 30S and 50S, respectively), the aa-tRNA participates in rapid peptide bond formation. A perfect match between codon and anticodon not only stabilizes A-site tRNA binding at both stages of decoding but also promotes GTPase activation and aa-tRNA accommodation (10–13).

**Figure 1.**
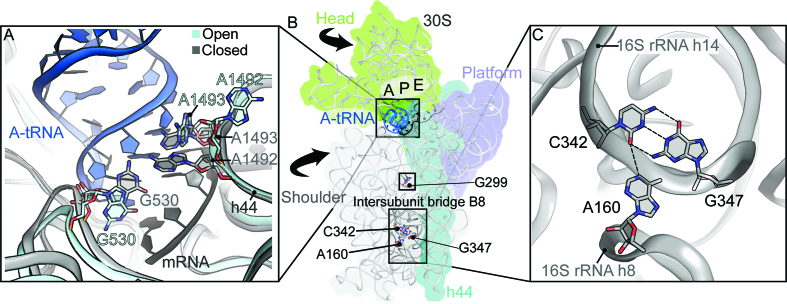
Location of G299A and G347U *ram* mutations on the 30S subunit. (**A**) Zoomed in view of the 30S A site including the A-site tRNA and mRNA codon. The open (PDB code 4V6G) compared to the closed form (PDB code 4V5L) of the 30S, showing differences in the positioning of 16S rRNA nts A1492, A1493 and G530. (**B**) Overview of the 30S as viewed from the intersubunit space (with the 50S removed) with the 30S head, shoulder and platform domains, h44, A-, P-, and E-tRNA binding sites, and A-tRNA indicated. Arrows indicate rotations of the head and shoulder seen upon A-tRNA binding. The upper box denotes the A-tRNA, the middle box indicates the location of G299 and the lower box defines the intersubunit bridge B8. (**C**) Base triple interaction between 16S rRNA nts C342 (helix h14), G347 (helix h14) and A160 (helix h8) that is disrupted in the G299A and G347U *ram* mutations and upon EF-Tu•GTP•aa-tRNA binding.

The ribosome plays an active role in aa-tRNA selection. Binding of aa-tRNA to the 30S A site causes a local rearrangement of 16S rRNA nts as well as global conformational changes in the 30S subunit (Figure 1). Universally-conserved nts G530, A1492 and A1493 reposition to monitor codon–anticodon pairing (14,15) (Figure 1A). A1492 and A1493 flip from 16S rRNA helix 44 (h44) to dock into the minor groove of the codon–anticodon helix while G530 rotates from a *syn* to *anti* conformation to form an interaction with A1492. This is accompanied by ‘downward’ movement of the 30S head and ‘inward’ rotation of the 30S shoulder (Figure 1B), movements collectively termed *domain closure*. These changes are proposed to be important for GTPase activation and productive aa-tRNA incorporation (14,16).

A number of mutations in the ribosome that increase miscoding cluster not only near the 30S A site but also to distal regions, consistent with a role for ribosome dynamics in the decoding process. These ribosomal ambiguity (*ram*) mutations generally increase the affinity of tRNA for the A site, stimulate GTPase activation during initial selection, and increase aa-tRNA misincorporation during the proofreading stage (17–22). The first studied *ram* mutations map to the interface of ribosomal proteins S4 and S5, proteins associated with the shoulder and platform domains, respectively (17). The location of the S4/S5 mutations strongly suggested that they perturb the dynamics of the shoulder relative to the platform. These mutations are believed to lower the energetic cost required for domain closure and thereby increase miscoding (17,23–26). While this model is attractive, direct evidence that *ram* mutations act by promoting domain closure has yet to be reported.

A screen for 16S rRNA *ram* mutations identified several at the interface of the shoulder and platform domains near S4/S5 (e.g. G299A in h12), along with many in helices h8 and h14 (e.g. G347U in h14) (19). Helices h8 and h14 interact with each other and contact 50S ribosomal proteins L14/L19 to form intersubunit bridge B8, proximal to the EF-Tu binding site. Disruption of B8 either by truncation of h8 (h8Δ3) or mutation G347U (in h14) results in high-level miscoding, indicating that B8 normally acts to negatively control the decoding process to ensure fidelity (19).

70S structures of mutant ribosomes harboring G299A or G347U reveal similar disruptions to B8 (21). Nucleotide G347 normally participates in a base triple interaction with C342 of h14 and A160 of h8 (Figure 1C). Mutation G347U disrupts this base triple and consequently B8. Mutation G299A is located in h12, ∼50 Å away from the 30S A site and ∼80 Å away from B8 (Figure 1). Remarkably, G299A also disrupts B8, suggesting that both *ram* mutations function through a similar mechanism. In the structures of 70S bound to EF-Tu•GTP•aa-tRNA ternary complex, B8 is also similarly disrupted (27–29). Based on these findings, it was proposed that GTPase activation normally involves disruption of B8, and the *ram* mutations reduce the energy barrier for GTPase activation by sterically or allosterically weakening B8 (21).

The crystallographic analysis indicated that G299A allosterically destabilizes B8, but whether this could fully explain G299A’s phenotype remained unclear (21). To address this question, Ying and Fredrick combined G299A with a truncation of h8 (h8Δ3), which ‘pre-disrupts’ B8, and measured the effects on miscoding (30). A moderate degree of positive epistasis was observed, suggesting that G299A acts partly via B8 and partly through another mechanism. Both G299A and G347U increase the affinity of tRNA for the 30S A site, consistent with conformational coupling between the decoding center, the 30S shoulder domain and B8 (30).

Here, we solved X-ray crystal structures of these mutant 70S ribosomes in the absence of an A-site tRNA and in the presence of a near-cognate A-site anticodon stem-loop (ASL). Both mutant ribosomes adopt the closed (on) state when either cognate or near-cognate tRNA occupies the A site. In the absence of A-site tRNA, the mutant ribosomes exhibit a partially closed (on) conformation, with the 30S shoulder rotated inward. G299A additionally causes G530 to rotate into the *anti* conformation, while G347U causes disruption of B8. Thus, both these *ram* mutations promote the open (off) to closed (on) transition, albeit in somewhat distinct ways.

## MATERIALS AND METHODS

### Structures of *Thermus thermophilus* 70S G299A and G347U ribosomes

Construction of the *Thermus thermophilus* 70S G229A and G347U strains, ribosome purification and crystallization were performed as previously described (19,21,31). Briefly, 70S ribosomes (4.4 μM) were programmed with mRNA (8.8 μM) for 6 min at 37°C. Five molar excess of tRNA^fMet^ (22 μM) and three molar excess of ASL^Leu^ (13.2 μM) were individually incubated for 30 min at 37°C (Supplementary Table S2). Deoxy BigCHAP (Hampton Research; 2.8 μM) was added just prior to crystallization. Crystals were grown by sitting-drop vapor diffusion in 4–5% polyethylene glycol (PEG) 20K, 4–5% PEG 550MME, 0.1 M Tris–acetate pH 7.0, 0.2 M KSCN and 10 mM MgCl_2_, and cryoprotected by increasing PEG 550MME in a stepwise manner to a final concentration of 30%. Crystals were flash frozen in liquid nitrogen for data collection.

X-ray diffraction data were collected at either the Southeast Regional Collaborative Access Team (SER-CAT) 22-ID beamline line or the Northeastern Collaborative Access Team (NE-CAT) ID24-C or ID24-E beamlines at the Advanced Photon Source (APS). Data were integrated and scaled using the program XDS (32). The structure was solved by molecular replacement in PHENIX (33) followed by iterative rounds of manual building in Coot (34) (Supplementary Table S1). All figures were prepared in PyMOL (35).

## RESULTS

### Disruption of intersubunit bridge B8 in mutant ribosomes containing either cognate or near-cognate A-tRNA

Previous structures of 70S-G299A and 70S-G347U ribosomes with cognate A-site ASL showed a disruption of bridge B8 (21). Mutation G347U prevents a triple base pair between G347U, C342 (both from h14) and A160 (h8) (Figure 1C). This mutation additionally causes a widening of h14 and a larger distance between h8 and h14, yet both h8/h14 helices move inward as part of 30S shoulder domain ‘closure’. A similar disruption of B8 was caused by G299A, despite that the mutation lies ∼80 Å from the bridge (21).

**Figure 2.**
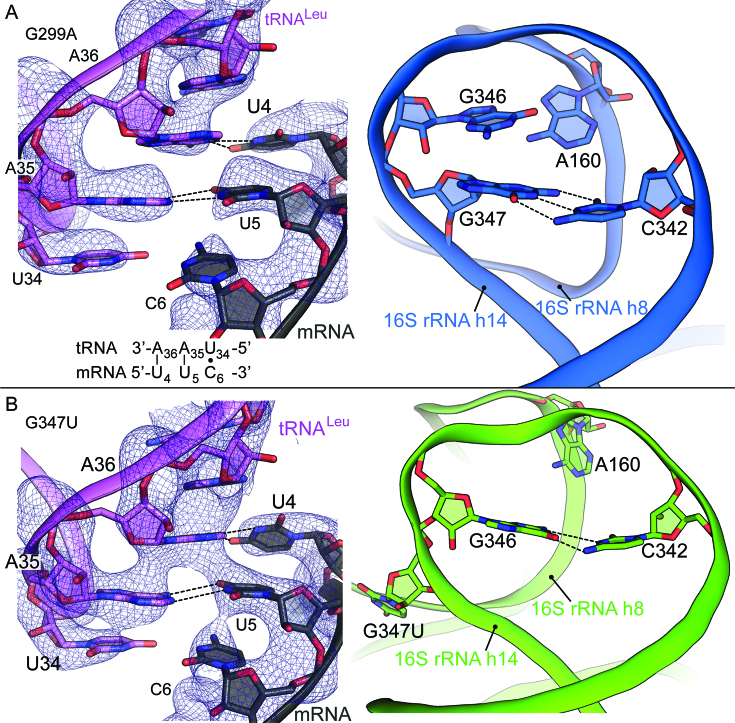
A-site near-cognate mRNA-tRNA interactions in the context of 70S *ram* mutations. (**A**) 70S-G299A *ram* ribosome containing a tRNA^Leu^ decoding the near-cognate Phe codon (5′-UUC-3′) in the A site (left). 2*F*_o_ – *F*_c_ density is shown at 1.5σ. In the right panel, a zoomed in view of the 16S rRNA helices h8-h14 interaction which is part of the intersubunit bridge B8. Helix h8 moves away from helix h14 ablating the base triple interaction between G347, C342 and A160 however preserving the G347–C342 basepair. (**B**) 70S-G347A *ram* ribosome containing a tRNA^Leu^ decoding the near-cognate Phe codon (5′-UUC-3′) in the A site (left). 2*F*_o_ – *F*_c_ density is shown at 1.5σ. In the right panel, a zoomed in view of the 16S rRNA helices h8–h14 interaction. The G347U mutation causes the nt to flip from the loop and a new interaction forms between G346–C342. Helix h8 moves away from helix h14 ablating the base triple interaction typically observed.

Those published structures contained ASL^Phe^ paired to the cognate 5′-UUC-3′ codon (all codons shown in the 5′ to 3′ direction) (21). Here, we solved a 3.7-Å structure of the 70S-G299A ribosome containing ASL^Leu^ (anticodon: 5′-UAA-3′) paired to UUC, that is, with a C–U mismatch at the third (wobble) position of the codon–anticodon helix (Figure 2A; Supplementary Table S1; Supplementary Figure S1). Watson–Crick interactions form between the first and the second positions of the codon–anticodon interaction (U4-A36 and U5-A35; mRNA-anticodon nts; mRNA nts numbered as +1 starting with the P-site mRNA codon). At the wobble position, the distance between U34 and C6 is too great to allow any hydrogen bonding (Figure 2A). Monitoring nts A1492, A1493 and G530 rearrange to dock into the minor groove of the codon–anticodon helix, with inward rotation of the 30S shoulder domain towards the 50S subunit and the 30S platform (Figure 1B). The base triple interaction between 16S rRNA nts A160, C342 and G347 is disrupted due to h14 widening (Figure 2A). In the previously reported structure of 70S-G299A containing cognate A-site ASL, there is a single hydrogen bond between the C5 keto group of G347 and the N3 position of C342 (21). In the corresponding near-cognate structure, G347 and C342 form a canonical Watson–Crick base pair similar to that observed in wild-type 70S (Figure 2A) (15). Overall, the structures of 70S-G299A mutant ribosomes bound by either cognate or near-cognate ASL closely resemble each other.

We next solved a 3.7-Å structure of the 70S-G347U ribosome with near-cognate ASL^Leu^ (anticodon: 5′-UAA-3′) paired to UUC (Figure 2B; Supplementary Table S1; Supplementary Figure S1). As observed in the analogous 70S-G299A structure (Figure 2B), Watson–Crick interactions form between the first and the second positions (U4-A36 and U5-A35) of the codon–anticodon helix but no base pair interactions occur at the wobble position because the distance between the nts is too great (Figure 2B). There is a slight widening of h14 coupled with the movement of h8 away from h14 prevents formation of the base triple that normally links helices h14 and h8 (Figure 2B). Interestingly, the electron density map reveals that the substituted nt (U347) is ejected from its usual position in the loop (Figure 2B; Supplementary Figures S1 and S2). This remodeling reduces the h14 loop size to three nts, allowing G346 to form basepair interactions with C342. This new basepair is analogous to the wild-type C342-G347 basepair into which A160 normally docks. However, in this 70S-G347U structure, h8 moves away from h14 and thus A160 is too distant to interact (Figure 2B). The absence of interactions between h8 and h14 is similar to that observed previously with 70S-G347U ribosomes containing cognate ASL^Phe^ (21).

This new 70S-G347U structure exhibited higher quality electron density than the earlier structures, allowing us to observe the reduced h14 loop size (Supplementary Figures S2 and S3). Hence, we revisited the h14 build in previous ribosome *ram* structures (21). We found that U347 is ejected from h14 in the previous 70S-G347U structure containing cognate A-site mRNA-ASL pairs (21), whereas G347 is retained in h14 in the 70S-G299A structure (Supplementary Figure S3). In other words, both 70S-G347U structures exhibit the reduced h14 loop size, regardless of whether cognate or near-cognate tRNA occupies the A site.

### Mutations G299A and G347U promote shoulder rotation in the absence of A-tRNA

**Figure 3.**
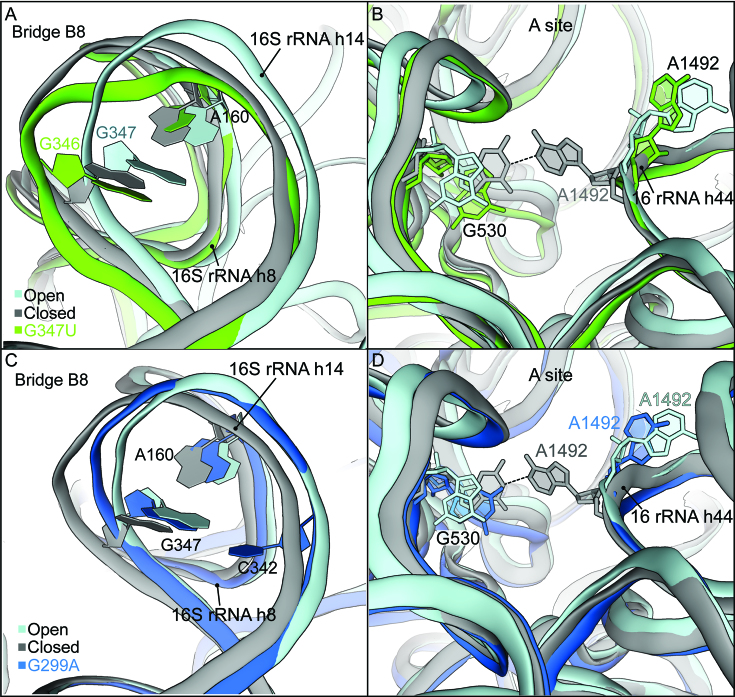
70S-G347U and G299A *ram* structure lacking A-site tRNA. (**A**) Zoomed in view of the 16S rRNA h8- h14 interaction (intersubunit bridge B8) in the 70S-G347U structure compared to open and closed 16S rRNA (PDB codes 4V6G and 4V5L, respectively). (**B**) Zoomed in region of the 30S A site comparing the 70S-G347U structure with open and closed form of the 16S rRNA. G530 and A1492 for each structure are highlighted as sticks. (**C**) Zoomed in view of the 16S rRNA h8–h14 interaction (intersubunit bridge B8) in the 70S-G299A structure compared to open and closed 16S rRNA (PDBs used are the same as in Figure 1A). (**D**) Zoomed in region of the 30S A site comparing G299A rRNA with open and closed form of the 16S rRNA. G530 and A1492 for each structure are highlighted as sticks.

To better understand how each of the *ram* mutations impact ribosome conformation, we solved structures of programmed ribosomes containing P-site tRNA^fMet^ and lacking A-site tRNA. In the 70S-G347U complex, determined at 3.2-Å resolution (Figure 3, Supplementary Table S1), B8 is disrupted, with h8 too distant from h14 to form the triple base pair (Figure 3A). Additionally, the 30S shoulder domain is rotated inward, which brings G530 closer to A1492 of h44 (Figure 3B). In other words, mutation G347U *alone* primes the ribosome for tRNA acceptance by shifting the conformational equilibrium of the ribosome toward the closed (on) state. Although G530 is closer to A1492, it does not undergo the *syn* to *anti* conformational change that normally accompanies A-site tRNA binding (14). The 70S-G299A complex lacking A-tRNA was solved to 3.5 Å resolution (Figure 3C, D; Supplementary Table S1). This structure, unlike all the other *ram* structures hitherto described, shows an intact B8, with formation of the A160-C342-G347 base triple (Figure 3C). However, similar to the 70S-G347U empty A-site structure, the 30S shoulder domain of the G299A ribosome adopts the closed-state position. This movement of the shoulder domain positions G530 close to A1492 of h44, and in this case, G530 adopts an *anti* conformation, poised to monitor the codon–anticodon helix (Figure 3D) (13,14). Thus, both mutant ribosomes exhibit a partially closed (on) conformation in the absence of A-tRNA. Mutation G299A promotes inward shoulder rotation and *syn*-to-*anti* isomerization of G530, while G347U promotes inward shoulder rotation and disruption of B8.

## DISCUSSION

**Figure 4.**
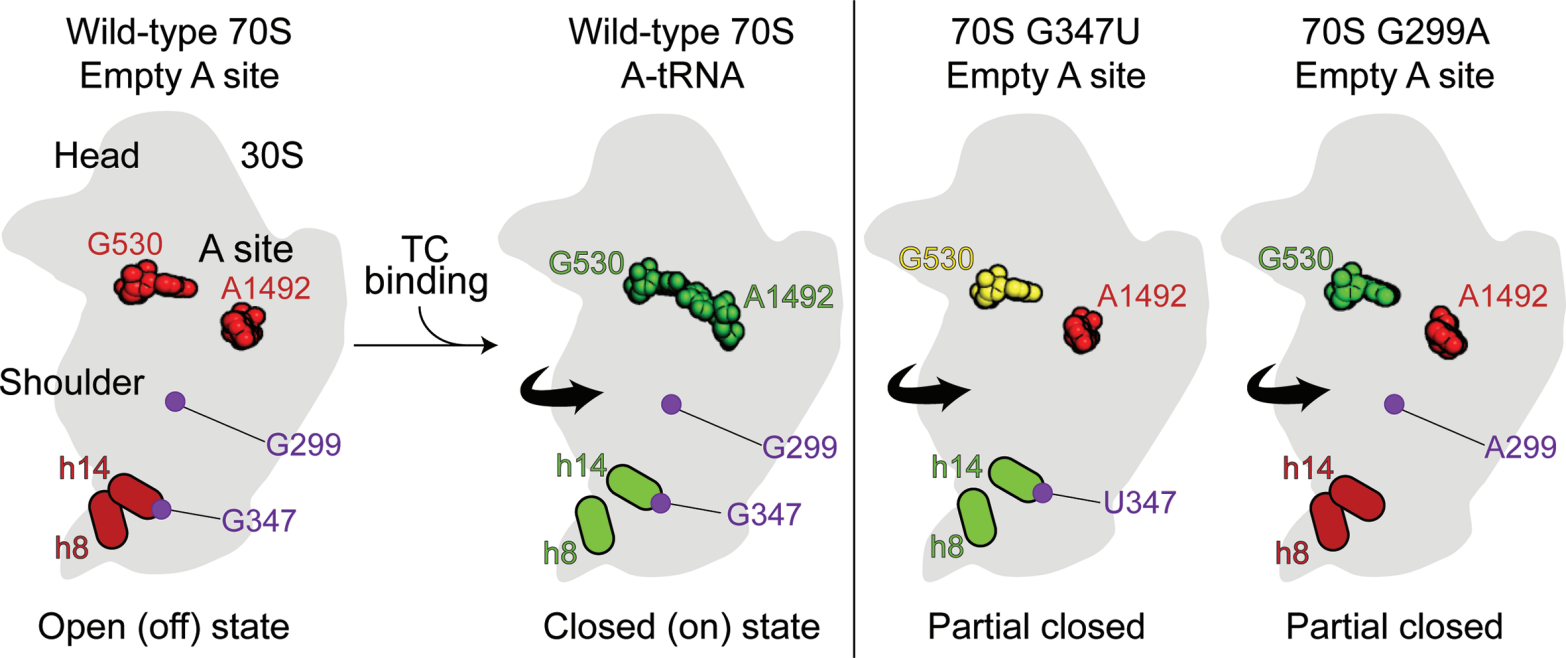
Model for how 70S *ram* mutations G299A and G347U alter decoding. Comparison of wild-type 70S with an empty A site (**A**) (PDB code 4V6G; (40)), wild-type 70S with a cognate A-site tRNA bound (**B**) (PDB code 4V51; (15)), 70S G347U *ram* mutation (**C**) and 70S G299A *ram* mutations (**D**) both in the absence of A-site ligands (this study). G530, A1492, helices h8 and h14 are colored green, red or yellow to denote closed (‘on’), open (‘off'), or Partial closed (‘semi-on’) positions, respectively.

The mechanism of decoding relies on a conformational change in the ribosome that occurs upon A-tRNA binding. A1492, A1493 and G530 rearrange to interact with the codon–anticodon helix in the decoding center, a change associated with more global motions of the 30S subunit (14). Previous genetic and structural studies of 16S *ram* mutations suggested that 30S shoulder rotation and bridge B8 disruption were key aspects of this open (off) to closed (on) transition (19,21,22,30). Here, we provide the first direct evidence that *ram* mutations promote inward 30S shoulder rotation, indicating the functional importance of this motion in the decoding mechanism. Mutation G299A additionally primes the decoding center, inducing rotation of G530 from *syn* to *anti* (Figure 3D; Figure 4). In the presence of A-tRNA, G299A allosterically promotes disruption of bridge B8 (21), the main target of *ram* mutations in the 16S rRNA (19,22). Bridge B8 is compromised or disrupted in the 70S-G347U ribosomes lacking A-site tRNA (Figure 3A), in 70S-G299A and 70S-G347U ribosomes in the presence of A-tRNA (cognate or near-cognate; (21), Figure 3B,D), and in wild-type ribosomes bound by EF-Tu ternary complex (27–29). Collectively, these findings provide compelling evidence that both 30S shoulder rotation and B8 disruption are key aspects of the open (off) to closed (on) transition.

Ogle and Ramakrishnan first proposed that domain closure played an important role in decoding, based on their structural studies of the 30S subunit (36). They observed that codon–anticodon pairing in the A site coincided with inward rotations of both the 30S head and 30S shoulder. In the context of the 70S ribosome, we have found no significant differences in the position of the 30S head domain among the various complexes compared. This suggests that the functionally relevant motion is rotation of the shoulder rather than rotation of the head.

Like *ram* mutations, aminoglycosides containing the 2-deoxystreptamine core cause miscoding. These compounds bind h44 in a way that occludes A1492 and A1493, causing these nts to adopt ‘flipped out’ conformations. It has been proposed that aminoglycoside binding pays part of the energetic cost of the A-site rearrangement that normally occurs upon codon recognition (37). In this manner, aminoglycosides promote the open (off) to closed (on) transition and thereby increase misincorporation rates. Recent kinetic studies by Ehrenberg and coworkers lend strong support to this model (38). Their data suggest that, in a four-step scheme of initial selection, aminoglycosides promote the third step, attributed to A-site rearrangement/ domain closure, whereas high Mg^2+^ concentration additionally influences earlier steps. We propose that *ram* mutations act much like aminoglycosides to promote the open (off) to closed (on) transition. In line with this view, these mutations reduce the fidelity of initial selection without increasing *k*_cat_/*K*_M_ in the cognate case (22).

How does the open-to-closed transition activate the GTPase (G) domain of EF-Tu? A recent cryo-EM study provides a simple answer to this long-standing question (39). Korostelev and coworkers determined structures of ribosomes bound by cognate or near-cognate EF-Tu•GTP•aa-tRNA in different conformations. Ribosome complexes in the closed state (i.e. with A-site nts fully docked into the codon–anticodon helix and the 30S shoulder rotated inward) showed direct interaction between the G domain of EF-Tu and the sarcin–ricin loop (SRL) of the 50S subunit. Other complexes, which had an unrotated shoulder domain and A-site nts either undocked or partially docked, showed considerable distance between the G domain and SRL. The proportion of complexes in the closed state was much larger when cognate ternary complex was used. These findings suggest that cognate codon–anticodon pairing promotes the open-to-closed transition, and 30S shoulder rotation is needed to position G domain against the SRL, enabling GTPase activation.

Our current work increases the number of structures relevant to the mechanism of decoding, snapshots of potential intermediates in the process. Several structures (37,39), including those of Figure 3, can be described as ‘semi-on’ or ‘partially-closed’ and these structures differ with respect to one another. This is consistent with the view that A-site rearrangement, shoulder rotation, and B8 disruption are loosely coupled events (30), which probably occur in random order. These motions, which collectively define the open (‘off') to closed (‘on') transition, become favorable upon codon–anticodon pairing, leading to productive aa-tRNA incorporation.

## DATA AVAILABILITY

Coordinates and structure factors were deposited in Protein Data Bank under accession codes 6BUW, 6BZ6, 6BZ7 and 6BZ8.

## Supplementary Material

Supplementary Data
