# Supplementary material for: Ribosomal ambiguity (ram) mutations promote the open (off) to closed (on) transition and thereby increase miscoding
Source: Nucleic Acids Res. 2018 Nov 22;47(3):1557–63. doi: 10.1093/nar/gky1178 (PMC6379664; doi:10.1093/nar/gky1178)
Supplement: Supplementary Data [file gky1178_supplemental_files.pdf]

Supplementary Information for

Ribosomal ambiguity (ram) mutations promote the open (off) to closed (on) transition and thereby increase miscoding

Eric D. Hoffer[1], Tatsuya Maehigashi[1], Kurt Fredrick[2,*] and Christine M. Dunham[1,*]

[1]Department of Biochemistry, Emory Antibiotic Resistance Center, Emory University School of Medicine, Atlanta, Georgia, 30322

[2]Department of Microbiology and Center for RNA Biology, The Ohio State University, Columbus, Ohio, 43210

*To whom correspondence should be addressed:

Christine M. Dunham (Tel: 1-404-712-1756; Fax: 1-404- 727-2738; E-mail: christine.m.dunham@emory.edu)

Kurt Fredrick (Tel: 1-614-292-6679; Fax: 1-614-292-8120; E-mail: fredrick.5@osu.edu)

This PDF file includes:

- Supplementary Tables 1 to 2
- Supplementary Figures 1 to 3
- References for SI reference citations

SUPPLEMENTARY TABLES

Supplementary Table 1. Crystallographic data and structure statistics.

	G299A empty A	G299A ASL^{Leu}	G347U empty A	G347U ASL^{Leu}
Data collection				
Space group	$P2_12_12_1$	$P2_12_12_1$	$P2_12_12_1$	$P2_12_12_1$
Cell dimensions				
a, b, c, Å	210.2 449.4 618.5	210.7 446.1 616.0	209.7 448.0 618.8	210.8 448.8 618.4
α, β, γ, °	90.0 90.0 90.0	90.0 90.0 90.0	90.0 90.0 90.0	90.0 90.0 90.0
Resolution, Å	122.0-3.5 (3.6-3.5)*	126.7-3.7 (3.8-3.7)	124.5-3.3 (3.5-3.3)	134.7-3.7 (3.9-3.7)
R_{merge}, %	17.5 (85.7)	29.0 (98.2)	26.3 (141.4)	21.6 (100.4)
R_{pim}, %	10.6 (52.0)	11.7 (45.1)	9.9 (53.3)	6.3 (36.1)
I/σI	8.1 (1.6)	6.4 (1.5)	9.0 (1.7)	10.7 (2.1)
Completeness, %	97.9 (99.4)	99.3 (99.8)	99.4 (99.8)	99.9 (99.9)
Redundancy	3.4 (3.5)	6.9 (5.5)	7.8 (7.9)	12.5 (8.0)
CC 1/2	0.995 (0.375)	0.987 (0.384)	0.996 (0.428)	0.99 (0.681)
CC*	0.999 (0.739)	0.997 (0.745)	0.999 (0.774)	0.998 (0.900)
Refinement				
Resolution, Å	122.0-3.5	126.7-3.7	124.5-3.3	134.7-3.7
No. of reflections	714367	618526	829154	599523
R_{work}/R_{free}, %	22.3/26.3	24.8/28.8	23.8/25.7	22.1/23.8
No. of atoms	291969	295578	291771	295687
Rmsd				
Bond lengths, Å	0.004	0.003	0.009	0.010
Bond angles,°	0.92	0.81	1.04	1.15
PDB ID	6BUW	6BZ7	6BZ6	6BZ8

*Values in parentheses are for the highest-resolution shell

Table S2. RNA oligos used in this study

mRNA	5'- AGGGAUUUAAAAUCCCU -3'
ASL^{Leu}	5' - GGCAAGGAGGUAAAAAUGUUCCAGA-3'

SUPPLEMENTARY FIGURES

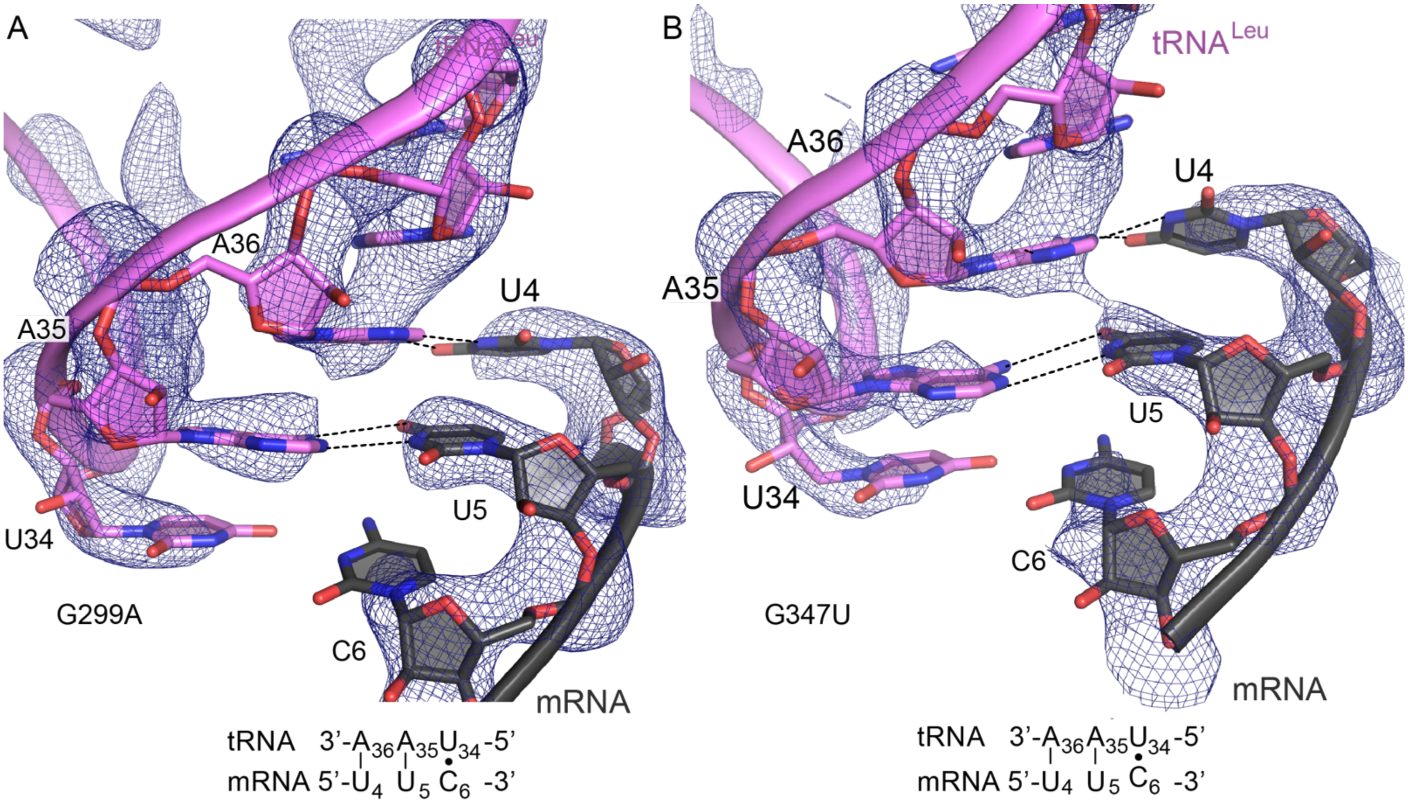

Supplementary Figure 1. Omit electron density maps of A-site near-cognate mRNA-tRNA interactions in the context of 70S *ram* mutations. (A) 70S-G299A *ram* ribosome containing a $tRNA^{Leu}$ decoding the near-cognate Phe codon (5'-UUC-3') in the A site. F_o-F_c composite omit density contoured at 2.0σ. (B) 70S-G347A *ram* ribosome containing a $tRNA^{Leu}$ decoding the near-cognate Phe codon (5'-UUC-3') in the A site. F_o-F_c composite omit density contoured at 2.0σ.

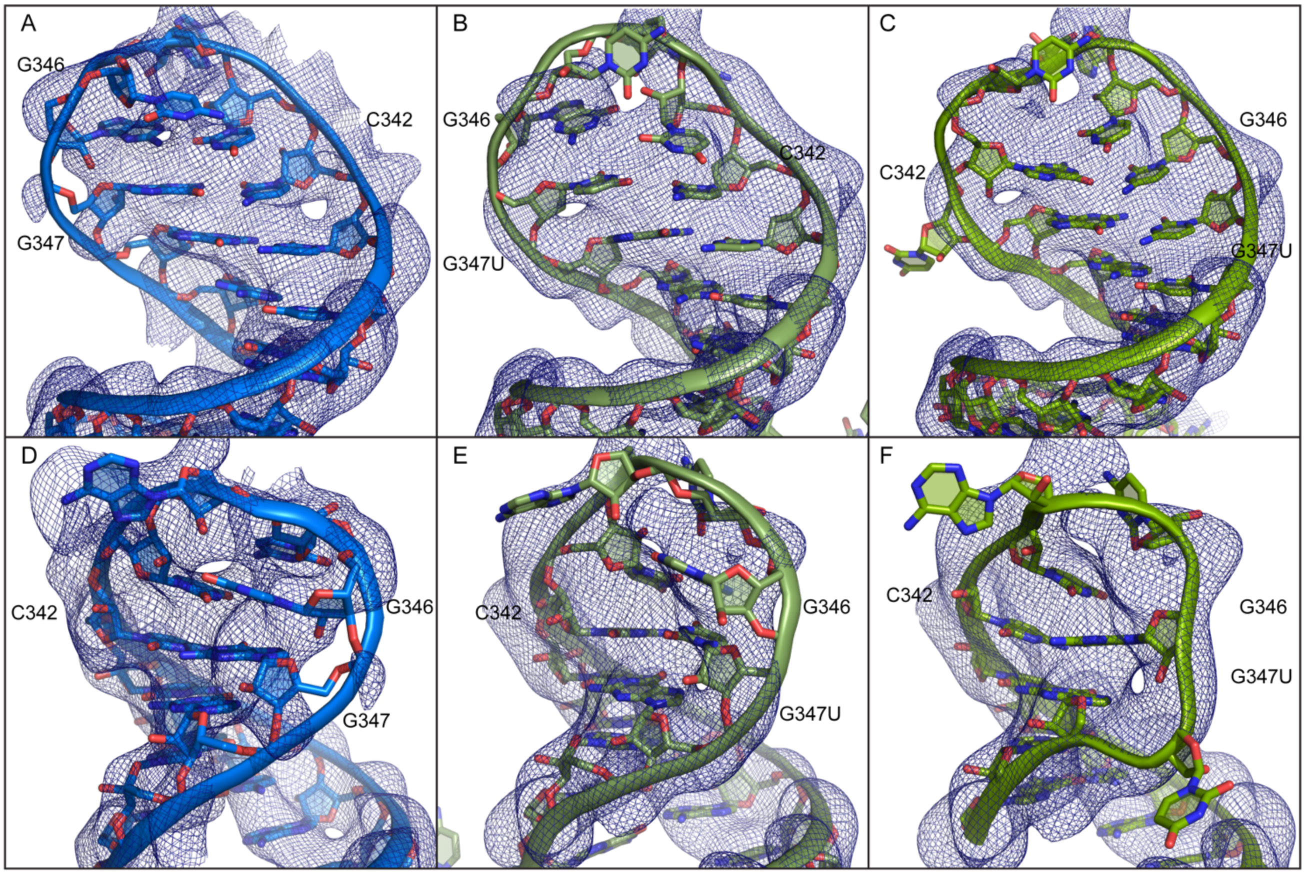

Supplementary Figure 2. Omit electron density maps. (A) 16S rRNA h14 in the 70S-G299A ribosome structure bound to near-cognate ASL^{Leu} (this study). The h14 models accurately fits the electron density. (B) h14 model from the 70S-G347U cognate mRNA-tRNA pair (PDB code 4V8J; (1)) placed in the 70S-G347U near-cognate mRNA-ASL density. (C) Rebuilt h14 model of the 70S-G347U near-cognate mRNA-ASL structure shown with F_o-F_c composite omit density contoured at 2.0σ. Panels D-F are 180° vertical rotations of panels A-C, respectively. All $2F_o$-F_c maps were contoured at 2.0σ.

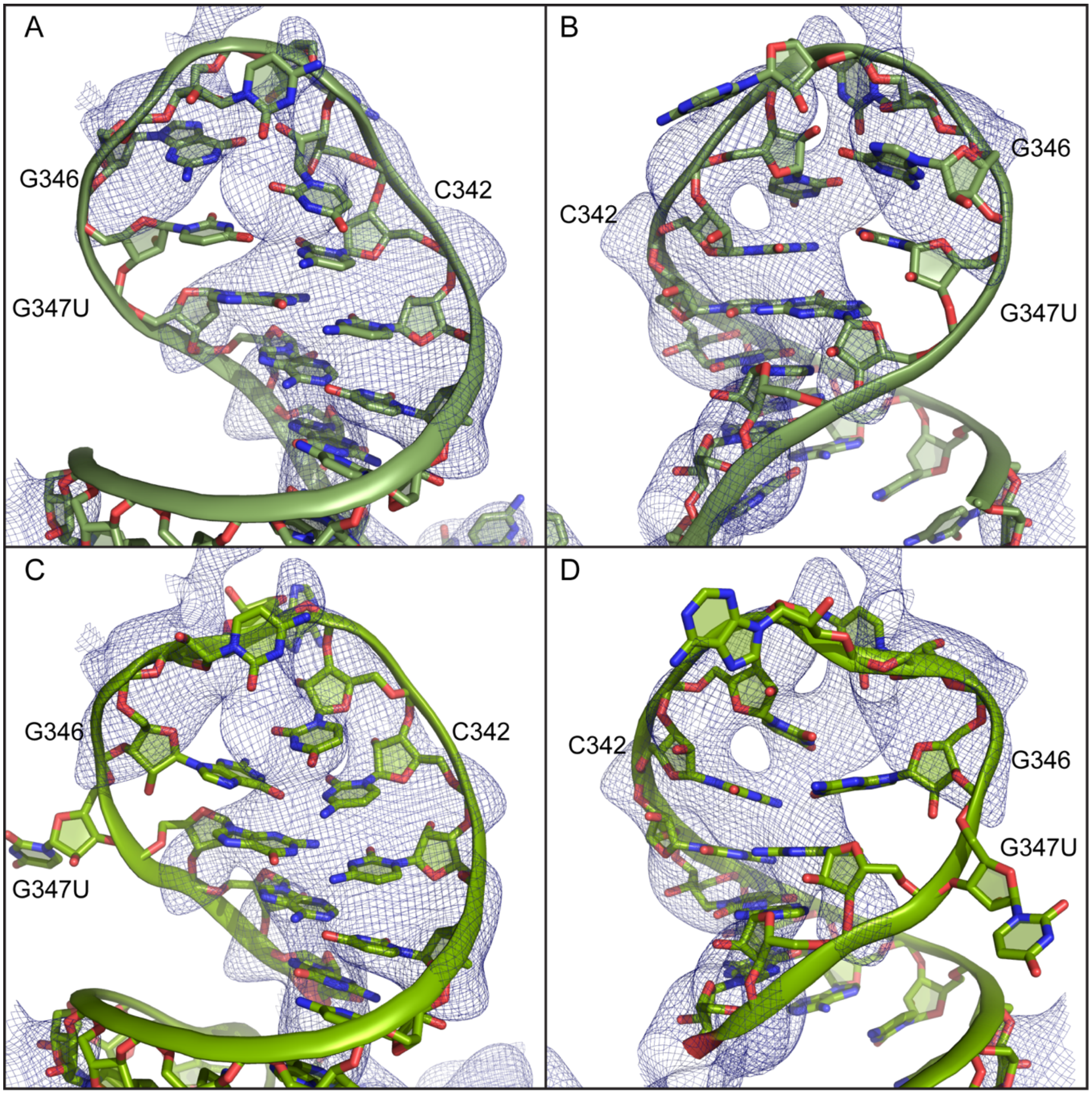

Supplementary Figure S3. Remodeling of h14. (A) 16S rRNA h14 model and electron density from the 70S-G347U cognate mRNA-tRNA pair (PDB code 4V8J; (1)) placed in the $2F_o$-F_c composite omit map contoured at 2.0σ. (B) A 180° vertical rotation of panel A. (C) Rebuild of 16S rRNA h14 (this study) placed in Fagan *et. al.*, 2013 (PDB code 4V8J) density (same $2F_o$-F_c composite omit map shown in panel A) emphasizing the better placement of the model. D) A 180° vertical rotation of panel C.

References

1. Fagan CE*, et al.* (2013) Reorganization of an intersubunit bridge induced by disparate 16S ribosomal ambiguity mutations mimics an EF-Tu-bound state. *Proc Natl Acad Sci U S A* 110(24):9716-9721.
